# Potential long-term effects of SARS-CoV-2 infection on the pulmonary vasculature: Multilayered cross-talks in the setting of coinfections and comorbidities

**DOI:** 10.1371/journal.ppat.1011063

**Published:** 2023-01-12

**Authors:** Rahul Kumar, Öznur Aktay-Cetin, Vaughn Craddock, Daniel Morales-Cano, Djuro Kosanovic, Angel Cogolludo, Francisco Perez-Vizcaino, Sergey Avdeev, Ashok Kumar, Anil Kumar Ram, Stuti Agarwal, Ananya Chakraborty, Rajkumar Savai, Vinicio de Jesus Perez, Brian B. Graham, Ghazwan Butrous, Navneet K. Dhillon

**Affiliations:** 1 Department of Medicine, University of California San Francisco, San Francisco, California, United States of America; 2 Lung Biology Center, Zuckerberg San Francisco General Hospital, San Francisco, California, United States of America; 3 Max Planck Institute for Heart and Lung Research, Member of the German Center for Lung Research (DZL), Member of the Cardio-Pulmonary Institute (CPI), Bad Nauheim, Germany; 4 Institute for Lung Health (ILH), Justus Liebig University, Giessen, Germany; 5 Division of Pulmonary and Critical Care Medicine, Department of Internal Medicine, University of Kansas Medical Center, Kansas City, Kansas, United States of America; 6 Centro Nacional de Investigaciones Cardiovasculares (CNIC), Madrid, Spain; 7 Department of Clinical Medicine, Aarhus University, Aarhus, Denmark; 8 Department of Pulmonology, I.M. Sechenov First Moscow State Medical University (Sechenov University), Moscow, Russia; 9 Department of Pharmacology and Toxicology, School of Medicine, Universidad Complutense de Madrid, Madrid, Spain; 10 Ciber Enfermedades Respiratorias (Ciberes), Spain; 11 Instituto de Investigación Sanitaria Gregorio Marañón (IISGM), Madrid, Spain; 12 Division of Pulmonary, Allergy, and Critical Care Medicine, Stanford University Medical Center, California, United States of America; 13 Department of Internal Medicine, Justus Liebig University Giessen, Member of the DZL, Member of CPI, Giessen, Germany; 14 Frankfurt Cancer Institute (FCI), Goethe University, Frankfurt am Main, Germany; 15 Cardiopulmonary Sciences, University of Kent, Canterbury, United Kingdom; NYU Langone Health, UNITED STATES

## Abstract

The Coronavirus Disease 2019 (COVID-19) caused by Severe Acute Respiratory Syndrome Coronavirus 2 (SARS-CoV-2) and its sublineages pose a new challenge to healthcare systems worldwide due to its ability to efficiently spread in immunized populations and its resistance to currently available therapies. COVID-19, although targeting primarily the respiratory system, is also now well established that later affects every organ in the body. Most importantly, despite the available therapy and vaccine-elicited protection, the long-term consequences of viral infection in breakthrough and asymptomatic individuals are areas of concern. In the past two years, investigators accumulated evidence on how the virus triggers our immune system and the molecular signals involved in the cross-talk between immune cells and structural cells in the pulmonary vasculature to drive pathological lung complications such as endothelial dysfunction and thrombosis. In the review, we emphasize recent updates on the pathophysiological inflammatory and immune responses associated with SARS-CoV-2 infection and their potential long-term consequences that may consequently lead to the development of pulmonary vascular diseases.

## 1. Introduction

Despite the significant progress that has been made in understanding the host–pathogen interaction with Severe Acute Respiratory Syndrome Coronavirus 2 (SARS-CoV-2), several critical questions remain unanswered, most of which are being actively addressed by ongoing preclinical and clinical studies. Perhaps foremost is identifying the genetic and environmental risk factors that result in symptomatic versus asymptomatic infection and a severe versus mild clinical course once symptomatic. Additionally, although some patients infected with SARS-CoV-2 recover after infection, many patients do not recover completely and continue having symptoms. The impact of post-acute sequelae of Coronavirus Disease 2019 (COVID-19) (PASC), also known as “Long-COVID-19,” “Long-term COVID-19,” or “Long-haul COVID-19,” is a primary concern and needs serious attention. This group of patients, even those described as “mild,” presents cardiovascular, neurologic, dermatologic, and pulmonary ailments. The abnormal lung functions are accompanied by persistent dyspnea, general neurological decay, smell and taste disturbances, and chronic fatigue [1].

The acute effects of SARS-CoV-2 infection including increased inflammation, alveolar damage, thrombosis, edema, and accumulation of cytotoxic molecules in lung vasculature may persist resulting in long-term complications in COVID-19-recovered patients [2,3]. A major concern is a persistent pathology of the pulmonary vasculature in long COVID. There is a possibility that we might see a higher prevalence of pulmonary hypertension (PH) cases and severe lung complications than previously reported based on the following *hypotheses*, which need to be investigated:

1. There may be persistently impaired physiological gas exchange due to damaged lung architecture including the vasculature.
2. Primed immune cells due to viral infection may change their phenotype to detect autoantigens and trigger autoimmune diseases, or there may be persistent viral RNA or proteins. For example, there may a shift towards a regulatory T cell phenotype, which can contribute to autoimmune disease [4,5].
3. Microthrombi that developed during acute infection may persist and propagate, or there may be the development of late thrombi due to the locally activated immune system or persistent lung damage.
4. COVID-19 patients with other coinfections, who live at high altitudes, or who have preexisting autoimmune or cardiovascular diseases may be at particular risk for developing PH long term [6,7].

This review will elaborate on these hypotheses and discuss the potential for the long-term development of pulmonary vascular disease (PVD) complications focusing on the COVID-19-triggered inflammatory pathways that damage the pulmonary vasculature network that might contribute to long COVID-19 pathology.

## 2. The acute pathophysiology of COVID-19

**Fig 1:**
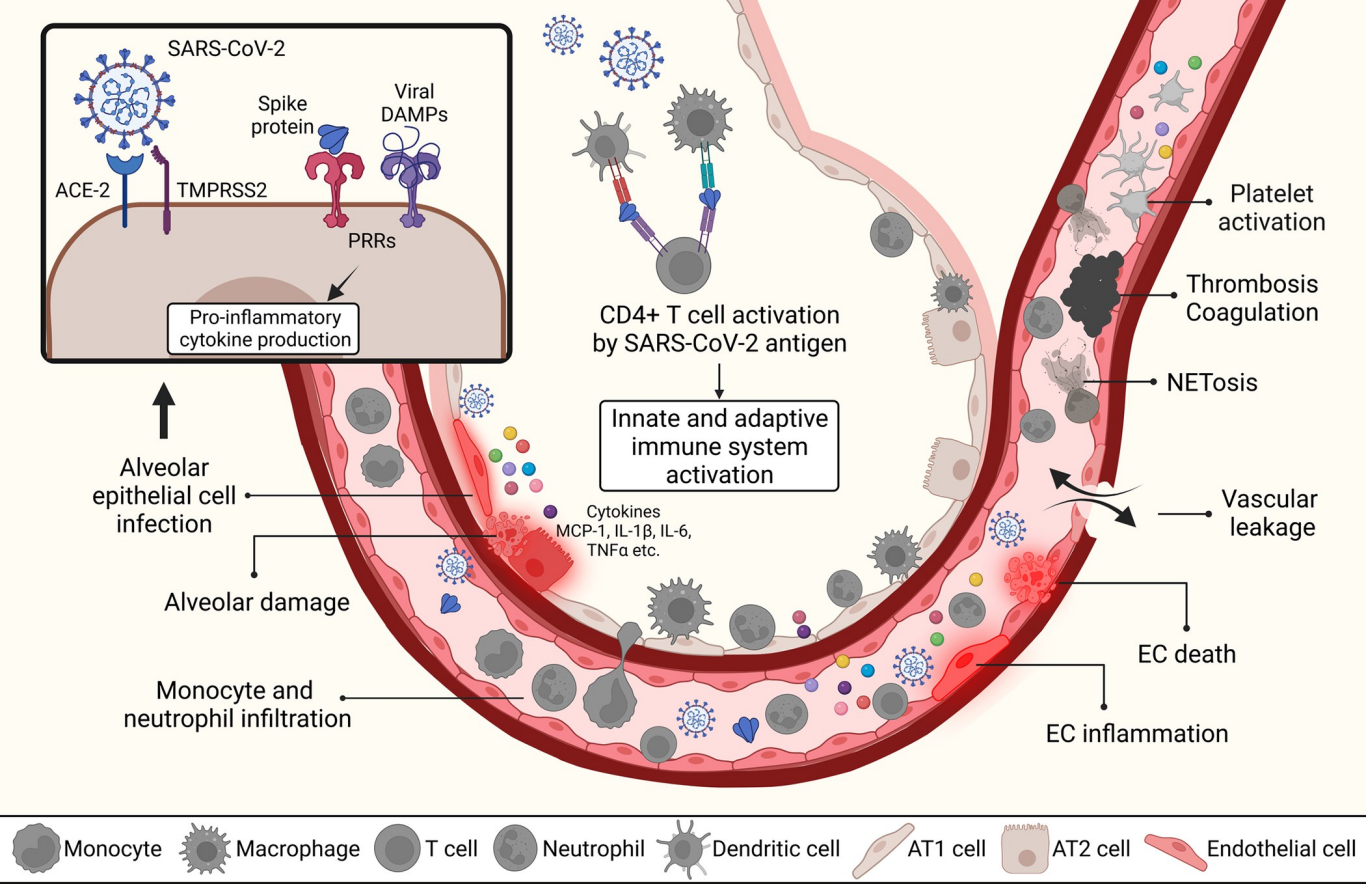
Acute response to SARS-CoV-2 infection in alveolar and vascular compartments. SARS-CoV-2 by utilizing ACE2 and TMPRSS2 invades epithelial layers to enter in alveolar compartment. Infected cell then releases DAMPs and PAMP molecules to alert immune system. While antigen presenting cells process and present SARS-CoV-2 antigen to CD4+ T cells, activation of innate and adaptive immune systems and production of inflammatory cytokines such as MCP-1, IL-1β, IL-6, and TNFα take place. These inflammatory monocytes cause production and activation of tissue factors that induce endothelial dysfunction. EC inflammation and cell death disrupts endothelial barrier and result in vascular leakage. Vascular neutrophilic inflammation can mediate platelet activation via proinflammatory cytokines to establish immune thrombosis. NET formation can contribute to prothrombotic state in hyperinflammation. Created with BioRender.com. ACE2, angiotensin converting enzyme 2; DAMP, damage-associated molecular pattern; IL, interleukin; PAMP, pathogen-associated molecular pattern; PRR, pattern recognition receptor; SARS-CoV-2, Severe Acute Respiratory Syndrome Coronavirus 2; TMPRSS2, transmembrane serine protease 2; TNF, tumor necrosis factor.

SARS-CoV-2 mainly targets respiratory tract and alveolar epithelial cells, expressing high levels of angiotensin-converting enzyme 2 (ACE2) receptors [8]. The ACE2 is the known cellular receptor and a necessary entry point for SARS-CoV-2 infection [8] via binding of Spike (S) surface glycoprotein (**Fig 1**). Heparan sulfate acts as a coreceptor enhancing the attachment of the virus [9]. S protein is cleaved by the host transmembrane serine protease 2 (TMPRSS2) and furin in a process known as S protein priming, essential for fusing the virus with the cell membrane [10]. After the invasion, the virus facilitates its multiplication in airway epithelial cells. Subsequently, infected host cells release damage-associated molecular patterns (DAMPs). These DAMPs and viral RNA are recognized by pattern recognition receptors (PRRs) on neighboring epithelial cells and other resident cells such as alveolar macrophages (AMs). Upon this recognition, inflammatory and antiviral effects are mediated via activation of the signal transducers and activators of transcription STAT 1-2/interferon regulatory factor (IRF) 9/interferon (IFN)-stimulated genes (ISGs) axis or by the activation of proinflammatory transcription factors such as nuclear factor kappa-light-chain-enhancer of activated B cells (NF-κB) that induces expression of tumor necrosis factor (TNF) α and interleukin (IL)-6 proinflammatory cytokines to generate a local inflammation for the recruitment of monocytes, macrophages, neutrophils, and lymphocytes. As programmed, resident and recruited immune cells are responsible for virus clearance, resolution, and recovery. However, when the immune system is hyperactivated and impaired releasing a wide range of proinflammatory cytokines and chemokines, it causes a pathological “cytokine storm” as observed in COVID-19 patients.

The resulting pathological components observed in the lung compartment include diffuse alveolar damage (characterized by type II pneumocyte hyperplasia, epithelial necrosis, fibrin deposition, and hyaline membrane formation [9–11]; edema; chronic interstitial inflammation; multinucleated giant cells [9,12]; and bronchopneumonia [11]). In addition, it has been established that COVID-19 pathology is associated with significant involvement of the vascular endothelial dysfunction [13]. Multiple histopathological reports show disruption of intercellular junctions, cell swelling, and a loss of contact with the basal membrane, with evidence of apoptosis, pyroptosis, and lymphocytic inflammation of the endothelium. In addition, vascular thrombosis associated with microangiopathy, occlusion of alveolar capillaries, and intussusceptive angiogenesis have been reported [14–16]. Further elevated D-dimers [17,18] and thromboelastogram data [19], together with higher rates of clinical deep vein thrombi and pulmonary emboli [20,21], have been documented as aberrant coagulation profiles. Thus, endothelial cells are essential contributors to the initiation and propagation of severe COVID-19 [22], although the detailed pathobiology is still unclear [17,23]. Endothelial cell injury may be indirect via circulating factors such as activated innate immune cells or thromboembolic or infection of adjacent epithelial or stromal cells. All these alveolar and vascular changes result in ventilation and perfusion mismatch [16,17,24–27].

### 2.1. Inflammatory and immunological response to SARS-CoV-2 infection

Many immune cells contribute to cytokine storm during the hyperinflammatory phase of COVID-19 as the accumulation of monocytes, macrophages, neutrophils, and T cells in the lungs was reported following SARS-CoV-2 infection [12]. Resident lung macrophages in the alveolar lumen known as AMs are the first line of defense against pathogens. Single-cell RNA sequencing analysis reported SARS-CoV-2 infection-mediated depletion of AMs and substituting those AMs with monocyte-derived macrophages in lung tissue of severe COVID-19 patients [28]. One recent study showed that M1 AMs accelerate viral spread since SARS-CoV-2 replication is facilitated in M1 macrophages effectively, whereas M2 AMs can restrict the spread [29]. Another report showed that CCL2 and CCL7 chemokines, which are responsible for the recruitment of macrophages, were elevated in bronchoalveolar fluid of severe COVID-19 patients [18]. On the other hand, infected circulating monocytes were also reported to contribute to inflammation [30]. Intermediate monocytes were found to release a high amount of IL-6 to improve pulmonary inflammation upon SARS-CoV-2 infection [30,31]. Consequently, monocytes and macrophages as highly plastic cells are crucial in SARS-CoV-2 infection, and it is essential to investigate their multisided effects to understand the pulmonary disease pathology.

Cytotoxic activation of CD4+ and CD8+ T cells and immunological memory help with antiviral immunity and clear the virus. In SARS-CoV-2 infection, T cell responses can be defined as a double-edged sword. On one hand, defective T cell response can contribute to inflammation and tissue damage, which worsens the outcomes of the disease [31]. On the other hand, T cell loss (lymphopenia) is a condition observed in SARS-CoV-2 infection and is associated with disease severity [32]. In severe cases of COVID-19, both CD4+ and CD8+ T cell numbers were reduced compared to moderate cases [33–36]. Upon viral infection, the innate immune system augments the expression of type I IFN to prime adaptive immune system. However, in COVID-19, impaired and delayed IFN responses correlate to disease severity [37,38]. Additionally, Yang and colleagues reported that CD8+ T cell differentiation is suppressed during prolonged SARS-CoV-2 positivity [39]. Imbalance between T cell responses and T cell differentiation mechanisms can be linked to continuous inflammation leading to abnormal overactivation of immune cells that might worsen the long-term outcomes in COVID-19-recovered individuals.

Neutrophils as other important contributors of cytokine storm were found elevated in the blood of COVID-19 patients. Neutrophils are recruited to pulmonary tissue upon hyperactivation of monocytes and macrophages in the lung. High neutrophil numbers in COVID-19 are positively correlated with disease severity, organ damage, and mortality [40]. Recruited neutrophils then develop neutrophilic vascular inflammation and immunothrombosis [41]. Neutrophil extracellular traps (NETs), which are formed through a biological process called NETosis (neutrophil death), have emerged as a key player in mediating inflammatory response [41–44]. NETs have antiviral effects but they can also accelerate the inflammation process [41]. Therefore, protective behaviors of neutrophils can be rapidly converted into a dysfunctional phenotype to exacerbate the disease progression [41,43].

IL-6 and TNF-α are major cytokines in the case of hyperinflammation, and they are mainly released by macrophages and lymphocytes. In a recent paper, patients with severe COVID-19 and high O_2_ requirements exhibited marked increases in circulating levels of extracellular vesicles (EVs), a lipid membrane–enclosed vesicular structures that carry a variety of cellular cargo such as lipids, nucleic acids, and proteins for cellular communications [45]. EVs rich in IL-6 and TNF family cytokines, the receptor for advanced glycation end products (RAGE), extracellular newly identified RAGE-binding protein (EN-RAGE), and programmed death-ligand 1 (PD-L1) are associated with disease severity [46]. EV-associated PD-L1 could contribute to hyperinflammation as well as to lymphopenia due to its interaction with PD-1, which induces anergy and apoptosis in T cells. Neutrophil markers including lectin-like oxidized low-density lipoprotein (LDL) receptor 1 and myeloproxidases were also found to be linked to EVs from the critically ill patients.

Besides the receptor function of ACE2 for SARS-CoV-2, its catalytic activity has important pathophysiological consequences for COVID-19 [47]. First, ACE2 converts angiotensin I into Ang1-9 and angiotensin II into Ang1-7, thereby negatively regulating the renin-angiotensin-aldosterone system (RAAS), promoting vasodilation and antioxidant and anti-inflammatory effects [48]. Secondly, ACE2 degrades des-Arg^9^-bradykinin and prevents the subsequent release of proinflammatory chemokines and exaggerated lung injury [49]. However, during the SARS-CoVs infection, ACE2 is down-regulated, which is expected to result in a loss of its protective anti-inflammatory effects and exacerbated vascular injury and pneumonia progression [47]. The interaction of the Spike protein can cause a marked reduction in the membrane-bound form of ACE2 both in cultured cells in vitro [50] and hamster lungs in vivo [51]. Additionally, ACE2 autoantibodies, which inhibit the catalytic activity of the enzyme, are increased in COVID-19 patients and may lead to enhanced proinflammatory responses and severity of COVID-19 outcome [52]. Taken together, these findings suggest that cytokine storm due to immune cell dysfunction in COVID-19 disease results in cellular and tissue damage. Signals from unhealed damage in tissue and organs might fuel continuous low-grade inflammation at local or systemic levels potentially translating into long-term post-COVID complications as discussed later in this review.

Several anti-inflammatory therapies have been clinically studied in COVID-19, including blocking the IL6-receptor (tocilizumab), Janus kinases (JAK) 1 and 2 (—barcitinib), and corticosteroids (dexamethasone—which is broadly immunosuppressant). The data on IL-6 receptor inhibition on acute endpoints in COVID-19 have been somewhat mixed, with no clear benefit in severe disease and a modest benefit in more mild disease (hospitalized and receiving continuous oxygen, but not requiring high flow nasal cannula or mechanical ventilation) [53,54]. Use of barcitinib to block JAK1/2, which are intracellular proinflammatory signaling mediators, has been more promising particularly in mild but hospitalized disease [55]. In contrast, dexamethasone was found to be of benefit in those with particularly severe COVID-19 [56]. On the basis of these data, current clinical guidelines advise barcitinib (or tocilizumab more modestly) for mild COVID-19, and dexamethasone in combination with barcitinib or tocilizumab for severe COVID-19. Importantly, all of these clinical data emphasize early endpoints (such as progression to severe disease, or mortality/ventilator free days out of the first few weeks) and do not assess the prevalence of PH at later time points.

### 2.2. Thromboinflammation and COVID-19

Thrombosis is a significant morbidity and mortality source for hospitalized COVID-19 patients and has been linked to hypoxia and organ failure [14,57,58]. Even after 160 years since its conception, Virchow’s triad is still a remarkable predictor of the location of clot initiation [59]. The broad vertices of the triad are (1) stasis (flow), (2) vessel wall injury (surface), and (3) hypercoagulability (blood constituents). In reality, these three factors are spatially and temporally highly interlaced. Flow stasis (vertex #1) is probably an established clinical risk factor for deep vein thrombosis (DVT) [60]. Slow flow (<100 s^−1^) in valve pockets also serves as a trigger for vessel wall injury (vertex #2), which also leads to accumulation of coagulation factors, and increases blood viscosity due to rouleaux formation (vertex #3) [61]. Overall, the Virchowian categorization is convenient for a reductionistic understanding of the complexity of COVID-19-associated coagulopathy.

Stasis will be contributed by myocardial injury, which is a relatively common feature of COVID-19 [62], and low cardiac output is an important complication in sepsis generally [63]. Venous thromboembolic events are much more common than arterial thromboembolic events among COVID-19 patients—the venous system is characterized by more sluggish blood flow than the arterial system, suggesting that stasis and not high shear rates, and hence secondary but not primary hemostasis, is the driver of clot initiation [64–66]. Regions of lung particularly injured by the viral infection and are hypoxic will have vasoconstriction resulting in locally decreased blood flow, which could also contribute to regional stasis.

Usually, quiescent endothelial surfaces are important hemostatic regulators. The anticoagulant functions on the endothelial surfaces include binding of thrombin to thrombomodulin; conversion of protein C to activated protein C; and clearance of activated coagulation factors and thrombin by antithrombin. In addition, the endothelium also regulates fibrinolysis by maintaining a balance between the production of tissue plasminogen activator that promotes fibrinolysis and plasminogen activator inhibitor that inhibits it [67]. However, damage to endothelial cells due to injury or inflammation may disrupt their hemostatic regulation leading them to adopt a procoagulant state that can lead to thrombotic events [68]. It has been shown that COVID-19 patients present with elevated levels of soluble thrombomodulin, von Willebrand Factor (VWF), and proinflammatory IL-6, all indicative of procoagulant endothelial phenotype, i.e., endotheliopathy [19,69,70]. Functionally, VWF facilitates platelet adhesion to the injured endothelial surface, triggering a primary hemostatic cascade. This, together with a relative decrease in VWF protease: ADAMTS-13 (A disintegrin and metalloproteinase with a thrombospondin type 1 motif, member 13) reported in COVID-19 patients, may result in larger VWF structures in circulation. These changes are only modest, which is consistent with thrombotic microangiopathy and not consumptive thrombocytopenia seen in COVID-19-associated coagulopathy [71,72].

The hypercoagulation and thrombosis in COVID-19-associated coagulopathy can be directly associated with dysregulated functions of immune cells due to cytokine storm [73]. The activation of the innate immune system, i.e., neutrophils and monocytes, modulates both intrinsic and extrinsic coagulation pathways. Upon activation, neutrophils and monocytes copiously release various inflammatory cytokines, including IL-8, TNF-α, and, most notably, IL-6, which has been associated as an indicator of mortality among severe COVID-19 patients [70,74]. IL-6 is a potent activator of platelets, and hyperactive platelets that display faster aggregation, showing increased spreading on both fibrinogen and collagen has been observed in COVID-19 patient plasma [75]. Neutrophils undergoing NETosis and forming NETs is emerging as an important contributor to immune thrombosis in COVID-19 [41–43] and may serve as a potential target to control coagulopathy. Their role in sepsis and acute respiratory distress syndrome (ARDS) pathogenesis is well documented, with NETs causing scattered microthrombi leading to multiorgan failure and death [76]. Platelet-leukocyte complexes have also been isolated from the circulation of COVID-19 patients. These complexes demonstrate an up-regulation in tissue factor expression, further contributing to the hypercoagulable state observed in COVID-19 patients [77]. In addition, endothelial cell adhesion molecules are up-regulated by proinflammatory cytokines, which establish a prothrombotic state in the microvasculature. Circulating EVs loaded with tissue factor, VWF, and many other thrombosis and coagulopathy mediators from critically ill COVID-19 patients were observed to cause endothelial apoptosis [46]. Lastly, reduced ACE2 in response to SARS-CoV-2 infection promotes a prothrombotic state [78]. All these changes contribute to the high rate of thrombotic complications in acute COVID-19.

Anticoagulant therapy for COVID-19 has been studied in multiple clinical studies, focusing specifically on acute endpoints. In general, these studies have not found benefit in critically ill patients [79], but there is some positive signal in more mild (but still hospitalized) patients, indicating that anticoagulation may prevent progression to more severe disease [80,81]. However, the current clinical evidence and guidelines suggest that anticoagulant therapy should not be used in all patients with SARS-CoV-2 infection [82].

### 2.3. Pulmonary endothelial dysfunction

Under normal conditions, vascular endothelium is the crucial barrier between pulmonary circulation and the lung interstitium. It possesses a series of properties that control vascular tone, inflammation, oxidative stress, vascular permeability, and the structure of the vessels and also provide a crucial interface in host defenses as front line [83,84]. However, whether this is caused by direct viral infection of the endothelium or inflamed-induced endothelial activation remains controversial. Multiple studies have reported that endothelial cells from different vascular beds express the ACE2 receptor and TMPRSS2 protease, which are required for SARS-CoV-2 infection [85,86], allowing virus to theoretically infect pulmonary endothelial cells. According to a recent study by Joffre and colleagues [87], SARS-CoV-2 can proliferate inside of human microvascular endothelial cells (HMVECs) and, as a result, activate endothelial cells, causing them to release IL-6, PAI-1, VEGF, IL-8, and/or CCL-2, with some changes depending on the patient donor. This is in agreement with the presence of viral elements within endothelial cells in the lung tissues from patients who died of COVID-19 [17]. However, Schimmel and colleagues [88] discovered that SARS-CoV-2 infection of primary human endothelial cells cannot result in the production of infectious virions and contribute to viral amplification. Nonetheless, they reported that virus may enter endothelial cells or respond to infection in the neighboring pulmonary epithelial cells or viremia by inducing the production of proinflammatory cytokines and adhesion molecules. Furthermore, Perico and colleagues [89] investigated the effects of SARS-CoV-2-derived Spike S1 protein on HMVECs and discovered that Spike S1 treatment resulted in dose-dependent increases in ICAM-1 and vWF, as well as increased deposition of complement protein C3 on the surface of HMVECs when cells were treated with higher concentrations of Spike S1.

Previous research has shown that during CMV, HIV-1, or HSV-1 infections [90–93], EVs can transmit viral proteins and genetic material from infected cells to healthy cells. EVs may contain S protein or its fragments, according to recent research on spike-expressing cells and examination of circulating EVs from COVD-19 patients [94–96]. Further, it’s been demonstrated that EVs released by lung epithelial cells transduced with lentiviruses encoding SARS-CoV-2 proteins transfer the viral RNA to cardiomyocytes, up-regulating the expression of inflammatory genes in the recipient cells [97] and raising the possibility that EVs may play a role in the spread of SARS-CoV-2 genomic RNA to uninfected cells. Along similar lines, one study found low copy numbers of SARS-CoV-2 RNA in exosomes extracted from COVID-19 patient plasma [98], while a more recent investigation was unable to find the viral RNA in EVs circulating in hospitalized acutely infected COVID-19 patients [96]. Additionally, Dhillon lab has observed that the plasma-derived proinflammatory EVs isolated from COVID-19 patients induce the apoptosis of pulmonary microvascular endothelial cells and this effect increase with increasing disease severity [46]. Overall, endothelial damage or dysfunction brought on directly by SARS-CoV-2 virus or viral components exposure or indirectly as a result of a systemic inflammatory cytokine storm affects the vascular endothelium’s homeostatic function and therefore may serve many roles in determining or exacerbating the disease severity and mortality in COVID-19.

Dysfunction or injured endothelial cells can first lead to impairment of endothelial-dependent vasodilatation, causing ventilation-perfusion mismatch and hypoxemia, not only by a direct impairment of hypoxic pulmonary vasoconstriction but also acting as a trigger of the coagulation cascade, resulting in the coagulopathy observed in COVID-19 patients [13]. Second, loss of intravascular endothelial cells barrier integrity, characterized by the formation of intercellular gaps between endothelial cells due to cellular damage and apoptosis by SARS-CoV-2 virion or its components and/or proinflammatory cytokines [16,99], leads to increased vascular permeability. This subsequently leads to protein-rich edema fluid in airspaces, resulting in poor gas exchange and decreased blood O_2_ levels [13]. Macrophage-originated TNF-α regulates vascular integrity by remodeling endothelial junctions to enable the transmigration of more immune cells [100]. As another example of cytokine-induced endothelial dysfunction, Varga and colleagues correlated monocyte and neutrophilic infiltration in arterial vessels with the apoptosis of endothelial cells in a lung specimen from a COVID-19 patient [17]. Third, on the basis of the abovementioned cytokine storm, the exposure to proinflammatory cytokines such as IL-6, IL-1β, and TNF-α released by immune cells can cause up-regulation of endothelial cell adhesion molecules, which will result in the activated state of endothelial cells [101–103]. This activated endothelial cells in turn promote the recruitment, adhesion, and migration of activated monocytes and neutrophils [104], creating an amplification loop that keeps the cytokine storm going. Dysregulation of neutrophil, NK cells, and macrophage activity has been noted to correlate with increased vascular endothelial inflammation markers [105]. To generalize, cytokine storm-based prolonged inflammation and circulation of viral components in COVID-19 directly affects pulmonary vasculature homeostasis.

## 3. Potential long-term effects of COVID-19 on the development of PVD and pulmonary hypertension

Some of the pathological features of COVID-19 are the existence of so-called “silent hypoxia” and unusual and nontypical attributes of ARDS [106–108]. The “silent hypoxia” may occur during the COVID-19 pathology, and its severity is not directly linked with the respiratory problems experienced by the patients, or in other words, the presence of “silent hypoxia” does not initially provide the significant signs, like shortness of breath in COVID-19 patients [109,110]. In addition, the severe forms of COVID-19 are characterized by significant hypoxemia (arterial hypoxia) [111]. Recent papers demonstrated that patients with COVID-19 might develop ARDS and acute cor pulmonale [112,113]. Acute cor pulmonale occurs as instant and unexpected augmentation of the pulmonary vascular resistance, and it may appear due to the hypercapnia-associated pulmonary artery vasoconstriction [114]. According to echocardiography studies, 20% to 25% of acute COVID-19 cases report RV dysfunction, which could be linked to higher mortality [115–118]. Increased RV afterload can be secondary to pulmonary parenchymal abnormalities during acute infection, combined with macro- or micro-PVD as well as the negative inotropic, prothrombotic, and profibrotic effects of cytokine storm [119–121]. Additionally, patients with RV dysfunction and PH, in particular, experience worse clinical outcomes [117,122].

Regarding the chronic effects of SARS-CoV-2 infection and COVID-19 on pulmonary circulation and development of PH, our knowledge is still tabula rasa, but one may speculate this to be a potentially clinically relevant problem in coming years. According to a recent publication, the investigation of magnetic resonance imaging in COVID-19-recovered patients revealed reduced RV function [123]. Recent research from Romania revealed that 9.33% of post-acute COVID patients had PH [124]. An additional investigation using echocardiographic techniques estimated PH in 29.7% of COVID-19 patients with higher mortality [125], although right ventricular systolic pressures (RVSPs) decreased after 5 months in the patients that survived. As mentioned above, SARS-CoV-2 infection has been linked with injured endothelium, dysregulated inflammation, oxidative stress, and microthrombi [126,127], and these phenomena are also described in the pathology of PH. PH is a form of lung blood vessel disease characterized by inflammation, vasoconstriction, endothelial and smooth muscle proliferation, and dysregulated apoptosis. It has been well established that inflammation plays a key role in orchestrating PH pathophysiology [128,129]. Acute exposure with recently emerged corona virus SARS-CoV-2 and its variants shares key pathobiological signatures with PH including inflammation, alveolar epithelial damage, thrombosis, edema, and accumulation of cytotoxic molecules in the pulmonary vascular bed. Further infections and comorbidities contribute to the heterogeneity and pathophysiological consequences of PH and may promote the development of PVD in individuals with COVID-19 in years to come as hypothesized and discussed in detail in the following sections.

### 3.1. Chronic proinflammatory state and vascular injury

The development of pulmonary and cardiovascular manifestations of long COVID has been hypothesized as a consequence of increased IL-6 and TGFβ and reduced ACE2, promoting a chronic inflammatory, profibrotic, and hypercoagulable status [130,131]. During the acute phase of inflammation, anti-inflammatory factors from pulmonary structural or resident and recruited immune cells may not be sufficient to resolve the inflammation due to defects in immune response, and, therefore, full tissue repair cannot be accomplished due to unresolved inflammation. Persistent tissue damage caused by persistent inflammation in this case can be major fuel of continuous “chronic” inflammation. Endothelial damage-mediated vascular leakage is another result of SARS-CoV-2 infection-related impairment in the pulmonary vasculature. Allnoch and colleagues observed inflammatory infiltrates within the endothelial cell layer and reported endothelial inflammation-dependent vascular leakage in a SARS-CoV-2-infected golden Syrian hamster model [100]. IL-6 and TNF-α that are robustly high during COVID-19 have been linked to reduced BMPR2 expression [132] and associated with severe disease and death in patients with pulmonary arterial hypertension (PAH) [133–136]. Bone morphogenic protein (BMP) and transforming growth factor-beta (TGF-β) are known to synergistically activate regulatory T cells to reduce inflammation and prevent autoimmune disease. Therefore, a loss of bone morphogenetic protein receptor type 2 (BMPR2) function can result in dysregulated immune cell recruitment, increased cytokine expression, and vascular infiltration by inflammatory cells into the intima [132]. Loss of pericytes during severe cases of COVID-19 is associated with vascular inflammation [137,138]. Insufficient replenishment of pericytes can be hypothesized to address continuous vascular inflammation in post-COVID. With such examples, prolonged hyperinflammation-mediated irreversible damages and deficiencies in pulmonary vasculature upon SARS-CoV-2 infection might prevent anti-inflammatory-type immune cells and/or anti-inflammatory soluble mediators to transit from blood to inflaming tissue, and this might cause to develop chronic vascular inflammation, later involving in PVD pathogenesis, particularly PH [139].

Persistence of viral residues in lungs, blood, or other organs can also fuel inflammation at a certain degree, perhaps not at an exacerbated level but causing a continuous low grade of inflammation. Interestingly, components of SARS-CoV-2, namely Spike protein and viral RNA, have been reported to persist in circulation for up to 1 year or longer after acute SARS-CoV-2 infection in individuals with PASC symptoms [96,140]. Further, the effects of Spike protein on thrombosis and coagulopathy may be far reaching and highly significant, possibly in post-COVID syndromes as well, as in acute COVID-19.

In addition, it was reported that SARS-CoV-2 spike protein promotes hyperplasia and hypertrophy of vascular smooth muscle cells, which later might exacerbate cardiovascular outcomes [141]. Patterson and colleagues showed that SARS-CoV-2 S1 protein remained in CD16+ monocytes up to 15 months post-acute COVID-19 [142]. SARS-CoV-2 protein-expressing circulating monocytes might be directly related to prolonged inflammation, severity, and the long-term phase of COVID-19, since studies revealed the increase of intermediate (CD14+CD16+) and nonclassical (CD14^lo^CD16+) monocytes in COVID-19 [142]. One possible reason regarding viral particle persistence in post-sequelae pathology might be some deficiencies in protein degradation machineries upon SARS-CoV-2 infection, since SARS-CoV-2-induced autophagy dysregulations were reported [143,144]. These possible deficiencies might result in altered protein turnover rate, resulting in chronic presence of proinflammatory cytokines and viral proteins in circulation and tissues. Detection of viral RNA after SARS-CoV-2 infection has also been reported by multiple groups many months post-recovery from COVID-19 [2,3,96,145–147]. Possibly, there is an immune-escape strategy behind the persistence of undegraded RNA of SARS-CoV-2 like HIV where methylation of viral RNA was found to help avoid immune cell sensing [148,149]. Further studies will show whether SARS-CoV-2 virus pursues such an immune-escape strategy or not. On the other hand, chronic immune stimulation can also be dependent on the site of viral RNA persistence [149,150], leading to persistent symptoms with systemic inflammation versus remaining asymptomatic without fueling inflammation. Consequently, it is crucial to clarify drivers of viral persistence-regulated inflammation in long COVID.

Future studies regarding the involvement of EVs in long-term manifestations of COVID-19 will also be of high interest. EVs have been shown to contribute to vascular remodeling in PH and therefore may also contribute to the development of PH following SARS-CoV-2 infection. We previously reported EV-associated TGF-β1 as an important player in pulmonary vascular remodeling and fibrosis in PAH [46,151]. In addition, HIV-Nef-positive EVs are shown to contribute to the pulmonary vascular endothelial dysfunction [152]. It will be interesting to assess if EVs are involved in the long-term remodeling of alveolar and pulmonary vascular tissue as recent report from Dhillon lab suggests the presence of spike protein on small EVs circulating in individuals with long COVID or PASC [96].

Increased activity of the RAAS has been implicated in the etiology of PAH [153,154]. Augmented angiotensin II (Ang II) levels and Ang II type 1 receptor (ATR1) activation are also observed in experimental PAH and associated with disease progression and mortality in patients with idiopathic PAH [153]. RAAS inhibition attenuates PAH in experimental models [155], and it is associated with improved survival in veterans with PAH [156]. ACE2 mRNA expression in explanted lungs from patients with PAH was strongly (approximately 6-fold) and consistently up-regulated compared to controls in four independent transcriptomic studies and the meta-analysis [157]. Likewise, up-regulated ACE2 mRNA (4-fold) has also been reported in rat lungs with PH induced by monocrotaline [158]. However, lung ACE2 protein expression was decreased in PAH and several animal models of experimental PH [159]. This seems to be due to the oncoprotein murine double minute 2 (MDM2)-mediated ubiquitination of ACE2 leading to protein degradation [159] and possibly increased disintegrin and metalloproteinase domain-containing protein 17 (ADAM17) expression [157] leading to increased ACE2 shedding in these patients. The latter is consistent with increased ACE2 protein and TNF-α in plasma PAH patients [135,160]. The setting is even more complex because, despite increased plasma ACE2 protein, plasma ACE2 activity was reduced in these patients, possibly due to increased autoantibodies against ACE2 [160]. To summarize, reduced lung and plasma ACE2 activity should be interpreted as deleterious for PAH. In fact, the activation of pulmonary ACE2 with recombinant ACE2 has been proposed as a therapeutic strategy for both PAH [161] to counteract the pro-oxidant, proinflammatory, and vasoconstrictor effect of the RAAS [47]. The role of ACE2 in COVID-19 is complicated by its function as the critical host receptor mediating viral entry. Therefore, Spike protein mediated down-regulation of ACE2 during SARS-COV-2 infection or postinfection, and the resulting proinflammatory status may eliminate a protective mechanism in vulnerable immunocompromised predisposed patients and may potentially trigger the development of PAH.

In conclusion, the loss of barrier function in response to chronic inflammation and endothelial activation is expected to expose sub-endothelial tissue within arterial vessels, which includes smooth muscle cells, extracellular matrix proteins (for instance, collagen), and fibroblasts. Over time, serum factors may leak through the endothelial membrane barrier and activate pulmonary arterial smooth muscle cells (PASMCs), causing them to proliferate and release endogenous elastases that will break down connective tissue within the tunica media and adventitia. Consequently, release of fibrotic growth factors and other initiators of the vascular remodeling released by smooth muscle and adventitial cells in response may lead to the development of PH [162,163].

### 3.2. Comorbidities that may synergize with COVID-19 to predispose risks to pulmonary hypertension

#### 3.2.1. Heart failure

Heart failure (HF) is a known etiology of Group 2 PH, or PH due to left-sided heart disease [164]. Patients with HF are also shown to be at much higher risk of hospitalization and death during acute COVID-19 [165]. Therefore, it is reasonable to expect that COVID-19 may exacerbate HF without resolution or return to pre-COVID-19 function in some patients. In fact, COVID-19 itself increases the risk and burden of HF after 1-year post-acute infection, suggesting that the virus can cause long-term changes to cardiac function even in patients without preexisting heart conditions [166]. Furthermore, post-COVID patients are at significantly increased risk for developing dysrhythmias, which are known to adversely affect cardiac function and exacerbate HF [166–168].

Mechanisms of HF exacerbation in COVID-19 patients may be indirect or direct. Indirect pathways may damage cardiac tissue due to the propensity for viral infections to cause tachycardia, thus increasing the workload on the heart, or could be due to acute manifestations such as increased acidity of the blood, hyperinflammation, hypoxia, and/or increased reactive oxygen species (ROS) especially in patients with severe COVID-19 [169]. Direct damage to cardiac tissue may be caused by SARS-CoV-2 infecting cardiac cells. Cardiomyocytes in heart express ACE2, the primary receptor utilized by SARS-CoV-2 for infecting cells. Consequently, cardiomyocytes may be infected and damaged by SARS-CoV-2 [170]. If COVID-19 itself increases the risk for HF postinfection in patients without preexisting cardiac dysfunction, then acute or chronic clinical manifestations caused by SARS-CoV-2 may exacerbate cardiac dysfunction in patients with HF and cause irreversible cardiac tissue damage. If left ventricle function is decreased by these changes, then this will reduce its ability to efficiently pump blood into systemic circulation, resulting in increased pulmonary circulation pressure. Consequently, patients with chronic HF who have had COVID-19 may be put at further risk of developing PH secondary to left heart disease due to irreversible exacerbations of their preexisting condition and/or due to post-acute sequelae of COVID-19 that affects lung and cardiac function.

#### 3.2.2. Chronic lung diseases

Chronic lung diseases (CLDs), namely idiopathic pulmonary fibrosis (IPF) and chronic obstructive pulmonary disease (COPD), are already known etiologies of Group 3 PH [164]. One may hypothesize that acute SARS-CoV-2 infection in patients with CLD may further increase the risk of developing PH or lead to the development of other forms of PH, such as Group 1 PAH. Acute COVID-19 may also accelerate the development and/or progression of PH secondary to CLD. Here, we will focus on how IPF may predispose someone to develop or worsen preexisting PH following acute COVID-19.

**Fig 2:**
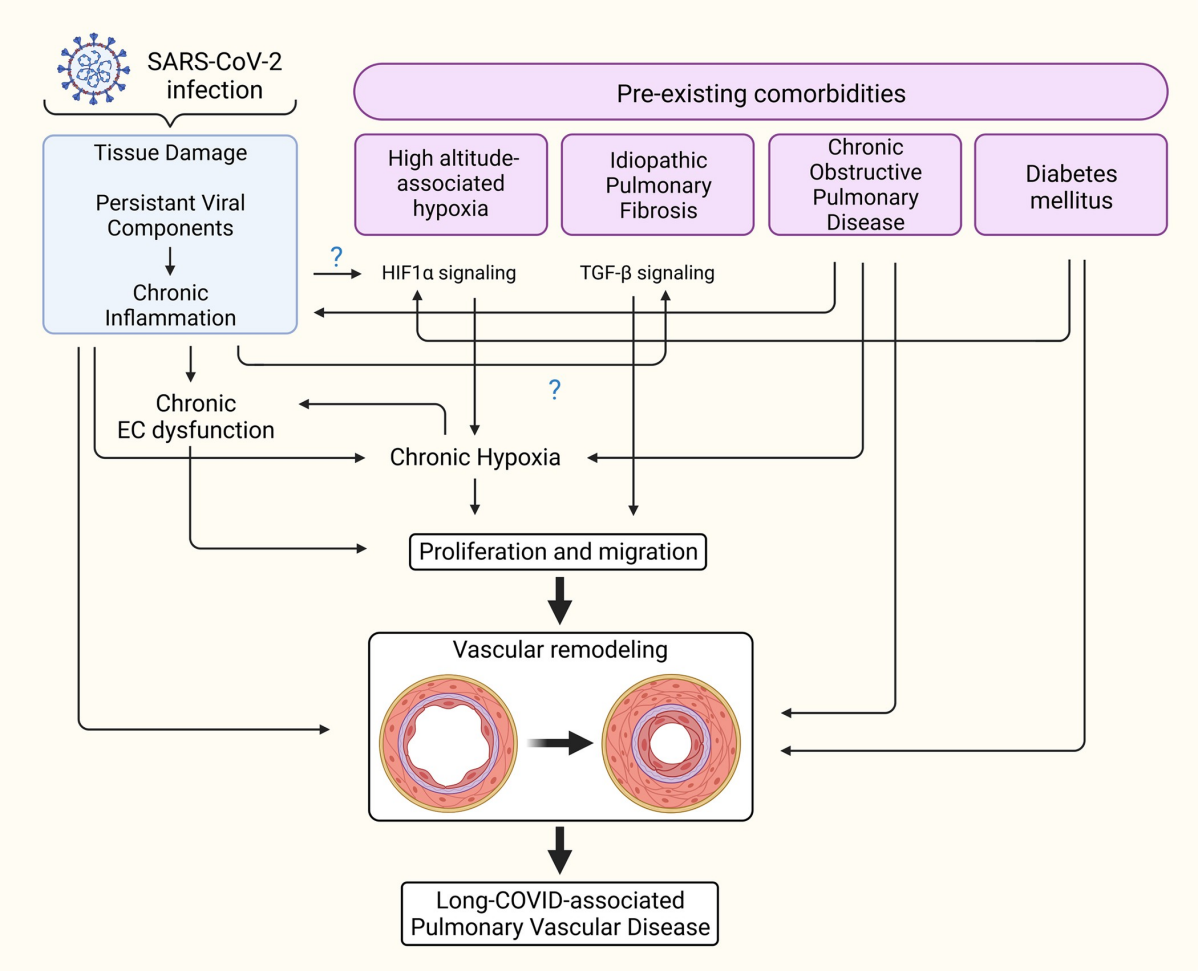
Comorbidities that may synergize with COVID-19 to predispose risks to PH. The possible interactions between long COVID state and preexisting comorbidities that may contribute to pulmonary vascular remodeling and the development of PH. Created with BioRender.com. COVID-19, Coronavirus Disease 2019; HIF1-α, hypoxia-inducible factor 1-alpha; PH, pulmonary hypertension; SARS-CoV-2, Severe Acute Respiratory Syndrome Coronavirus 2; TGF-β, transforming growth factor-beta.

**Pulmonary fibrosis**: PH is frequently found in concomitance with IPF, and the presence of both diseases in patients is correlated with worse clinical outcomes than either disease alone [171]. This is not surprising, as IPF and PH share some common pathways in their pathophysiology. In IPF, abnormal TGF-β signaling instigates pulmonary fibrosis and induces the expression of proteins that cause stiffening of interstitial lung tissue [172–174]. TGF-β is also a major actor in the development and progression of PH due to its involvement in pathogenic vascular remodeling [46,173,175] by inducing endothelial-to-mesenchymal transition (EndoMT) as well as PASMC proliferation and medial hypertrophy of arteries [175]. In IPF patients, TGF-β secreted by cells within the alveoli would theoretically be contained within the interstitial tissue of the lungs. In this case, TGF-β is primarily exerting its effects on cells within alveoli. It may be of interest to mention some of the new report that suggest potential increase in pulmonary fibrosis clinically due to COVID-19 [176,177]. Since COVID-19 can elicit an intense “cytokine storm” that promotes alveolar tissue damage, compromises alveolar epithelial integrity, and increases capillary permeability, then contents normally contained within the alveoli may leak into the bloodstream at a much higher level than normal, allowing for TGF-β in IPF patients to affect the pulmonary vasculature. This might predispose IPF patients who were hospitalized with severe acute COVID-19 to PAH. Interestingly, a study by Ferreira-Gomes and colleagues [178] demonstrated that TGF-β triggers a chronic immune reaction in patients who were hospitalized with COVID-19 for up to 60 days. Furthermore, some of the recent reports suggest potential increase in life-threatening and prolonged pulmonary fibrosis clinically due to COVID-19 [179–181]. SARS-CoV-2 infection increases the risk of some patients developing pulmonary fibrosis via TGF-β pathways [182]. These concerns suggest that TGF-β may originate from AMs/metaplastic type 2 alveolar epithelial cells and concurrent SARS-CoV-2 infection in patients with IPF, and the resultant damage to alveolar tissue from acute COVID-19 may allow for TGF-β to spill into the bloodstream for prolonged periods of time and instigate pulmonary vascular remodeling that can ultimately lead to the development of PAH (**Fig 2**).

**Pulmonary hypoxia**: It is well known that chronic pulmonary hypoxic conditions cause the development of pulmonary vascular remodeling and ultimately the occurrence of PH [183]. Pulmonary vascular remodeling that appears due to chronic and long-term exposure to high-altitude hypoxia is the key pathological characteristic of high-altitude PH (**Fig 2).** Studies suggest that the long-term stay or living at high altitude settings may induce the activation of the coagulation system and may lead to the prothrombotic state [184–190]. There may be interplay between high-altitude hypoxia and COVID-19 with its “silent hypoxia” and hypercapnia potentially responsible for augmenting the abnormal blood clotting associated with COVID-19. Therefore, one may speculate that high-altitude hypoxia would worsen the altered coagulation process in COVID-19 patients. However, multiple articles suggested that the pathogenesis of the novel coronavirus may be reduced at high altitudes and that people living there may be less susceptible to develop the severe form of COVID-19 [191–193]. Possible relevant reasons for such outcomes could be that a harsh high-altitude environment characterized by low humidity, increased UV radiation, and more ozone is not a favorable milieu for the survival of the virus [191,194]. Secondly, highlanders may possess some adaptations, such as the hypoxia-associated decrease in the expression of ACE-2, which is an entry point for the SARS-CoV-2. However, recent findings that there was a high rate of SARS-CoV-2 seropositivity among the highlanders in La Rinconada, Peru (the highest city in the world) suggested that there were no protective effects of altitude against the spread of the COVID-19 [195]. One study analyzed the mortality and COVID-19 in high-altitude geographic settlements and found a link between altitude and mortality due to the COVID-19 in males younger than 65 years [196]. More data are needed to support the “theory” that people living at high altitudes are protected against the SARS-CoV-2 [197–199].

#### 3.2.3. Diabetes mellitus

Patients with diabetes mellitus (DM) who contract infection with SARS-CoV-2 are at greater risk of developing severe COVID-19 with respiratory failure and in-hospital mortality [200,201]. Not only does COVID-19 put people with DM at-risk for poor prognosis during the acute disease course, but it also may serve as a trigger that accelerates molecular processes that are pathognomonic of vascular diseases, including PH. Patients with DM are already at increased risk of pulmonary emboli and PH due to hypercoagulability and increased frequency of atherosclerotic cardiovascular disease (ASCVD) [202]. This risk is exacerbated by COVID-19, which may mean that the concurrence of DM and acute SARS-CoV-2 infection, when put together, are major risks for the development of chronic thromboembolic pulmonary hypertension (CTEPH). Additionally, patients with both DM and PAH have lower 5-year survival rates compared with PAH patients without DM, indicating that diabetes contributes to PAH pathophysiology and risk of mortality [203]. We hypothesize that acute COVID-19, especially in severe cases, may instigate clotting and pulmonary vascular remodeling, which would predispose these patients to PH over time.

Despite sharing common pathways with PH pathophysiology and the apparent involvement of DM in PAH prognosis, diabetes by itself is not currently considered a direct etiology of PH. Nonetheless, endothelial dysfunction is a known complication of DM as exemplified by common adverse manifestations of DM including diabetic neuropathy, nephropathy, retinopathy, and slow healing wounds or ulcers [204,205]. Patients with DM have abnormal responses to hypoxia due to decreased baseline activity of hypoxia-inducible factor 1-alpha (HIF1-α), especially in those with poor glycemic control [206,207]. Normally, HIF1-α is important for tissue repair following ischemic vascular injury because it induces expression of growth factors, cytokines, and other chemoattractants important for recruiting endothelial progenitor cells (EPCs) to home within ischemic vascular tissue and maintain endothelial integrity [206,208]. In diabetic patients, this pathway of tissue repair is dysfunctional. As a result, diabetic patients with respiratory failure secondary to COVID-19 may be at risk for prolonged endothelial barrier dysfunction in pulmonary arteries due to lack of vessel tissue repair and consequently vascular remodeling.

Patients with DM may also have elevated activity of pathways that are also involved in PH pathophysiology. For instance, protein kinase C (PKC) expression is up-regulated in the vascular tissue of large arteries, renal glomeruli, and retina in DM, and its isoforms are shown to be involved in vascular complications of DM [205]. PKC is a regulator of smooth muscle contraction within the tunica media of arteries and can be induced by endothelin-1 (ET-1), which is also implicated in diabetic vascular complications [205,209]. Both PKC and ET-1 contribute to PH pathophysiology by inducing vasoconstriction within pulmonary arteries and thereby increase pulmonary vascular resistance and pressure. In fact, endothelin receptor antagonists are indicated as therapeutic agents for patients with Group 1 PH, or PAH, to antagonize continual vasoconstriction by these dysregulated pathways in PAH [209,210]. The PKC and ET-1 pathways may contribute to the risk of PH in DM who had COVID-19 due to the vasoconstriction caused by these molecules.

Lastly, diabetic patients are at increased risk of developing atherosclerosis due to increased levels of circulating lipids. COVID-19 may accelerate the formation of fibrotic atheromas and plaques within the pulmonary vasculature in predisposed patients, especially in patients with DM who are at increased risk of unresolved endothelial damage. If these plaques were to form in pulmonary vessels and occlude arteries to the point of hemodynamic significance, then this would increase mean pulmonary arterial pressure, pulmonary vascular resistance, and right ventricular afterload, the hallmarks of PH. Furthermore, COVID-19 induces a hypercoagulable state that also increases a person’s risk of clotting [131]. Atherosclerotic plaques increase the risk of these clots persisting long after acute COVID-19 and consequently may lead to long-term occlusion of pulmonary arteries consistent with CTEPH. Collectively, the processes described thus far may compound together in individuals with DM following acute COVID-19 and further increase the risk of development and progression of PH because of prolonged impaired vascular remodeling, unresolved clotting, and occlusion of pulmonary arteries.

### 3.3. COVID-19 in people living with HIV

**Fig 3:**
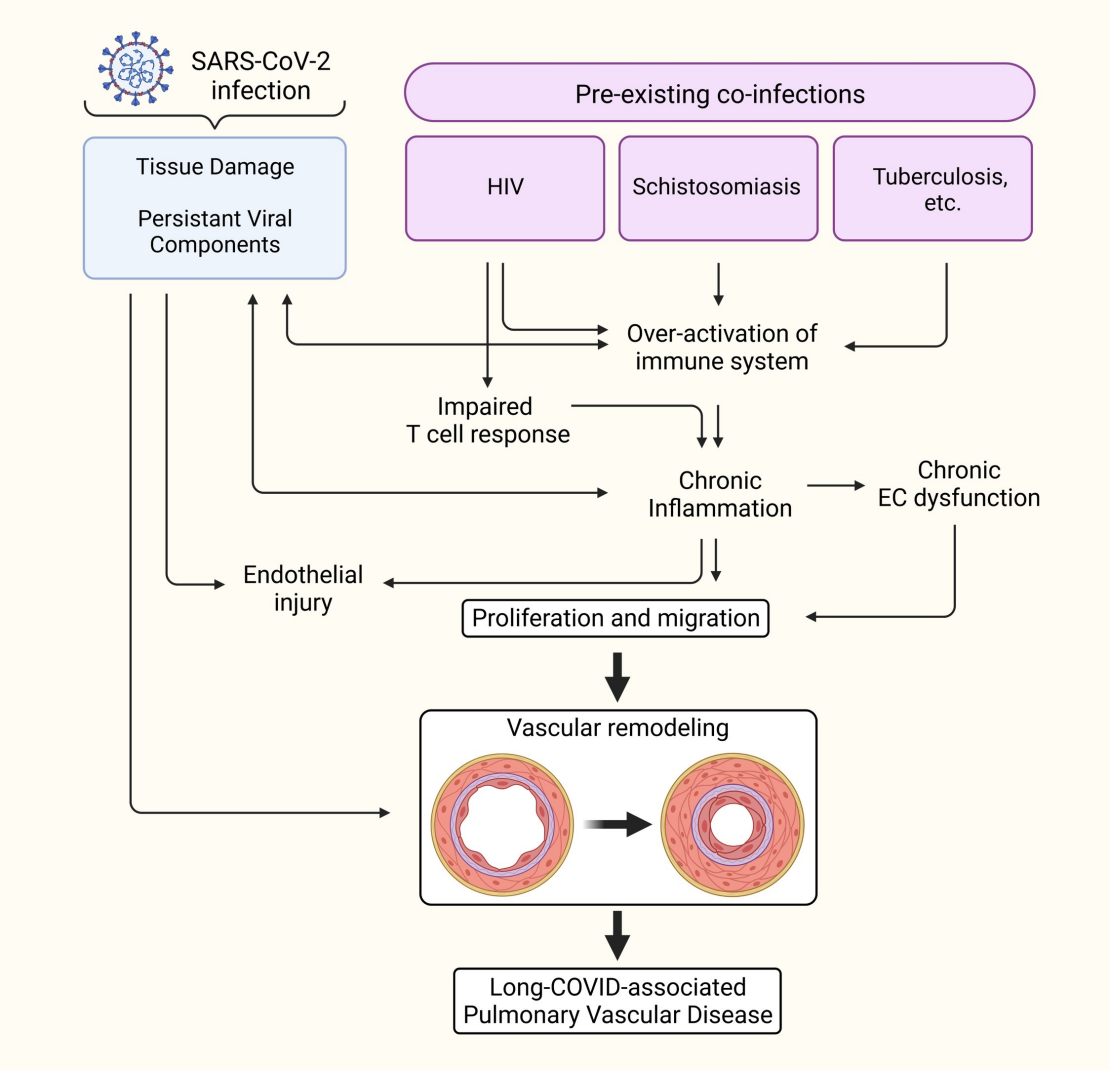
Coinfections that may synergize with COVID-19 to predispose risks to PH. Preexisting chronic infections can be speculated for their contribution to prolonged inflammation in long COVID state. Created with BioRender.com. COVID-19, Coronavirus Disease 2019; PH, pulmonary hypertension; SARS-CoV-2, Severe Acute Respiratory Syndrome Coronavirus 2.

COVID-19 in people living with HIV is linked to high risk of death by compared to people without HIV [211–213]. Other studies showed that persistent symptoms of post-acute sequelae of SARS-CoV-2 infection are more prevalent among HIV-infected individuals [214,215]. HIV causes chronic infection primarily in CD4+ T cells and is a definite cause of PAH [164,216]. Persistent SARS-CoV-2 infection, in theory, elicits chronic inflammation that may augment the endothelial dysfunction and vascular remodeling in people living with HIV-1 who are already predisposed to PH. Further, innate immune dysregulation does not just pose concerns for persistent SARS-CoV-2 infection and long COVID, but also reactivation of latent infections. A previous study conducted at the Gugulethu Community Health Centre Antiretroviral clinic in South Africa demonstrated that prior SARS-CoV-2 infection imposed a higher risk of impaired HIV viral control as measured by HIV viral load [217]. A duration for how long after SARS-CoV-2 infection this impaired HIV viral control might persist is not clear, but in conjunction with innate immune dysregulation, it’s possible that SARS-CoV-2 infection might allow for HIV provirus to be reactivated and replicate, increasing the risk for developing HIV-PAH initiated by HIV viral protein-mediated pulmonary vascular remodeling (**Fig 3**).

**Fig 4:**
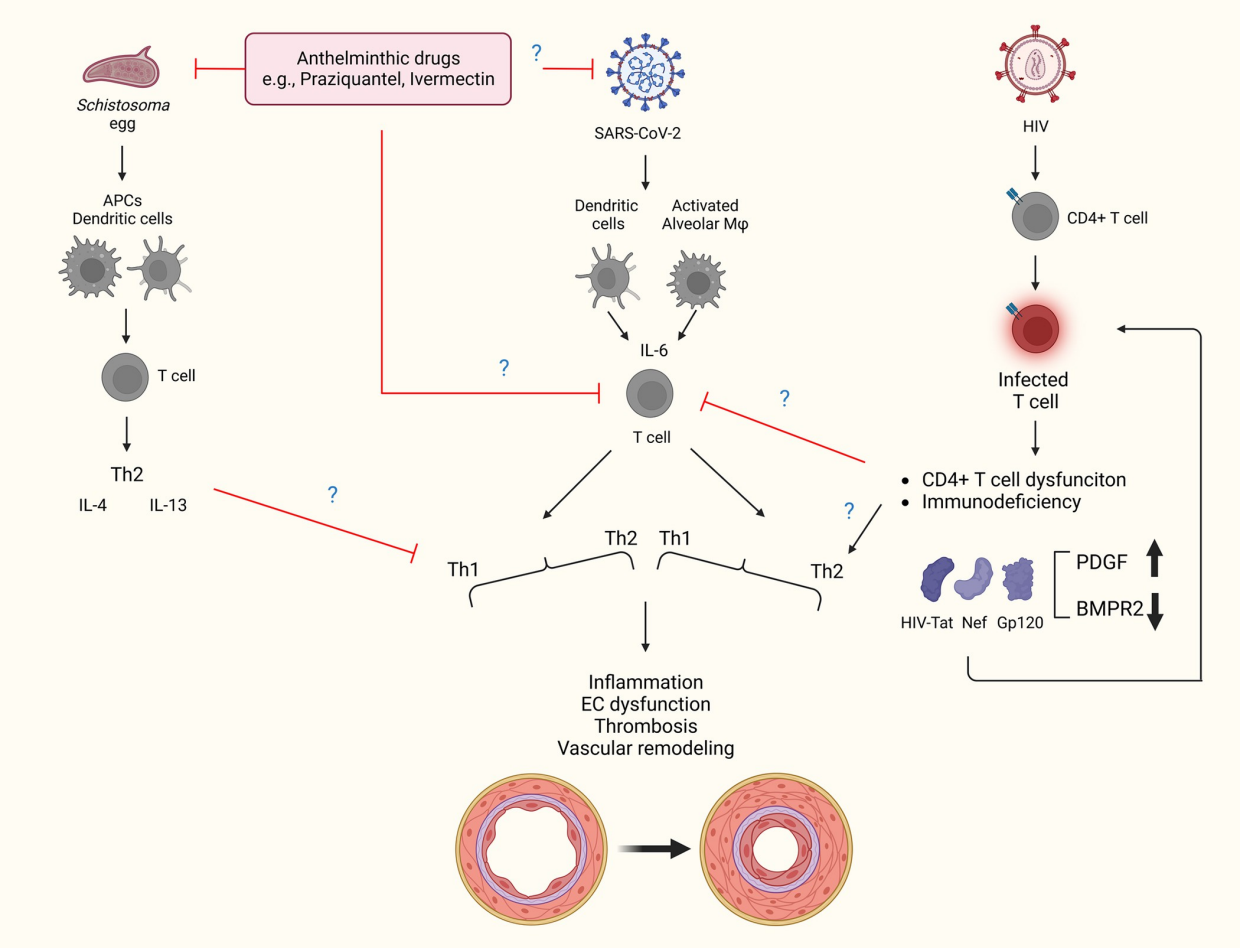
The schematic shows the activation of immune cells and cytokines trigged following *Schistosoma*, HIV, and SARS-CoV-2 infection. *Schistosoma* antigen is presented to dendritic cells that activates CD4 T cells to release proinflammatory IL-IL4 and IL-13 [129,252]. On the other hand, SARS-CoV-2 antigen is processed by dendritic and alveolar macrophages with the release of IL-6 cytokine that eventually favors Th1-induced cytokine storms [253,254]. *Schistosoma* or SARS-CoV-2 infection results into releases of Ly6c monocytes from the bone marrow compartment that drives inflammatory process [255,256]. Of note, coinfection with both *Schistosoma* and SARS-CoV-2 might lead to higher severe conditions. However, based on the recent report on less prevalence of COVID-19 in *Schistosoma*-endemic regions, there is a possibility that anthelminthic drugs such as praziquantel might have antiviral property to block the replication of SARS-CoV-2 or dampen the accumulation and production of IL-6, although this was not observed clinically for ivermectin [257,258]. Blockade of IL-6 might be helpful in mitigating cytokine storm and endothelial damage. The other possibility could be the counterbalancing of Th1-induced cytokine storm by the predominance of Th2 immunity due to prior *Schistosoma* infection. The overall effect of *Schistosoma* and SARS-CoV-2 coinfection on downstream pathological features such as endothelial (EC) dysfunction. HIV proteins are known to cause endothelial dysfunction and vascular remodeling [152,218,219] via skewing the Th1-Th2 balance. HIV viral proteins Tat, Nef, and gp-120 were reported to reduce BMPR2 expressions, while Tat and gp120 can induce expression of PDGF and involving in pulmonary vascular remodeling. Further detailed studies are required to answer whether preexisting chronic HIV infection is associated with prolonged inflammation in long COVID phase. Created with BioRender.com. BMPR2, bone morphogenetic protein receptor type 2; COVID-19, Coronavirus Disease 2019; IL, interleukin; PDGF, platelet-derived growth factor; SARS-CoV-2, Severe Acute Respiratory Syndrome Coronavirus 2.

Reactivation of HIV provirus allows for the expression of HIV proteins, such as HIV-Tat, -Nef, and -gp120. All three of these proteins are shown to cause endothelial dysfunction and apoptosis, initiating vascular remodeling [152,218,219]. It was also previously shown that Tat and gp120 can induce production of ROS and expression of platelet-derived growth factor (PDGF), which contribute to endothelial dysfunction and are involved in the pulmonary vascular remodeling seen in HIV-associated PAH [220]. A similar increase in apoptosis is seen in endothelial cells exposed to Spike protein [221] and circulating EVs from COVID-19 patients as mentioned above [46]. In HIV-associated PAH, the viral protein products Tat, Nef, and gp-120 have been shown to reduce BMPR2 expression [152]. It’s possible that these viral proteins contribute to BMPR2 suppression and endothelial injury upon reactivation of provirus in COVID-19 infection or in synergy with Spike protein that was recently reported to persist in circulation of individuals with long COVID [96] **(Fig 4)**. Like many viruses including SARS-CoV-2, HIV is capable of exhausting and suppressing T cell response to infection [222]. While it is clear that a monocyte–macrophage-driven Th1 response is activated early on in response to SARS-CoV-2 [46,223,224], patients who become critically ill mount an aggressive Th2 response as the Th1 response weakens [225]. HIV is known to dysregulate chemokine and cytokine cascades by skewing the Th1-Th2 balance in favor of a Th2 immune response to evade immune detection [226], a process that favors the development of PAH [227]. It remains to be seen whether coinfection with HIV and SARS-CoV-2 has a synergistic effect on immune dysfunction and clinical outcomes. Besides, preexisting chronic HIV infection can be speculated for prolonged inflammation in COVID-19-recovered patients. The inflammation status can be translated into hyperinflammation phase by coinfections.

### 3.4. COVID-19 in people living with schistosomiasis

Schistosomiasis is a parasitic infection that affects >200 million people and is a major cause of PAH worldwide. Currently, there are limited data and information about the potential impact of preexisting parasitic infections on risk of developing COVID-19 illness, and disease severity and long-term sequelae after becoming infected. Parasitic infections result in type 2 inflammation by the host immune system, characterized by cell populations including Th2 CD4 T cells, eosinophils and basophils, and cytokines such as IL-4, IL-5, and IL-13. In patients with chronic parasitic diseases such as schistosomiasis resulting in longstanding inflammation, these infections establish baseline type 2 immunity in the host. However, a robust type 1 host immune response is important to successfully suppress viral infections. Thus, a hypothesis is that patients with chronic parasitic infections will be relatively susceptible to infection by SARS-CoV-2. Consistent with this hypothesis are reports from African countries of a higher percentage of COVID-19 confirmed and active cases in schistosomiasis-endemic regions compared to nonendemic regions [228].

An alternative but not necessarily mutually exclusive hypothesis is that patients with chronic parasitic infections may be relatively protected from severe disease once having developed the initial infection. It is thought that a major mechanism of tissue injury in severe SARS-CoV-2 infection is excessive type 1 inflammation; a shift in the inflammatory “setpoint” prior to injury could thus decrease subsequent COVID-19 disease severity [21,229]. Further, Th2 cytokines down-regulate ACE2 expression, which could abrogate viral replication [230,231]. Consistent with this hypothesis, a group reported an inverse correlation between the number of COVID-19 cases and, in particular, the number of deaths in areas of endemic parasitic infections [232]. On a global scale, Africa (where many parasitic infections are endemic) contributes 0.88% cases and only 0.45% of total global deaths (despite having approximately 17% of the world’s population) [232]. In contrast, regions with lower prevalence of parasitic infections such as North America, Europe, and Asia accounted for the vast majority of COVID-19 cases and deaths [233]. A key limitation of these data is differences in testing and reporting from different parts of the world.

The antihelminthics praziquantel or ivermectin could also independently decrease Th1 inflammation. There may also be interindividual genetic variability in driving COVID-19-related severity among the populations of endemic and nonendemic regions, and SARS-CoV-2 itself may have different genotypes and phenotypes between regions or the world. There may also be mediators through different components of the immune system: for example, the complement system that bridges innate and adaptive immunity, and has been shown to be activated in both *Schistosoma* and COVID-19 infections, and thus may be another point of interaction. In addition, a large fraction of COVID-19 patients showed autoantibodies to multiple self-antigens, and these autoantibodies were correlated with the severity of the disease [234]. The presence of pathogenic autoantibodies had also been reported following *Schistosoma* infection [235]. Therefore, the cross-talk between these autoantibodies under the coexisting condition of schistosomiasis and COVID-19 would be another area to consider. Of note, a schematic has been presented (**Fig 4**) to simplify the possible hypothesis to explain the mechanism by which prior *Schistosoma* infection or use of anthelminthic drugs might attenuate the severity of SARS-CoV-2 in schistosomiasis-endemic regions. These hypotheses need to be tested and verified using animal models [236]. It should be noted that ivermectin was studied as an antiviral in multiple clinical trials of SARS-CoV-2-infected patients [237–239] and was uniformly found to not change acute outcomes such as progression to more severe disease. It is unlikely that many of the patients in these series were concurrently parasite infected as this was not a selection criterion (or prevalence assessed), so the effect of antihelminthics on Th1 versus Th2 bias in virus and parasite coinfected individuals has not yet been tested.

Overall, evaluation of these possibilities will depend on further epidemiological and demographic data on the coexistence of coinfections and COVID-19. Although studies are limited, more comprehensive research on coinfections with SARS-CoV-2 will help us to understand the role of comorbidities and coinfections on the development of chronic inflammation in post-COVID and inflammation outcomes that induce long COVID–associated PVD.

## 4. Future perspectives and conclusions

Overall, it appears highly likely that SARS-CoV-2 infection causes some degree of acute PVD as a major aspect of the acute pathology and that some pulmonary vascular injury is likely to be present in individuals with persistent symptoms, which has pathobiological aspects similar to PH. The interaction with preexisting PH or preexisting comorbidities and if this PVD slowly ameliorates or recurs late is yet to be determined. Further, the evaluation of the interactions between COVID-19 and other infections in the development of long-term PVD is warranted.

SARS-CoV-2 infects type II pneumocytes via ACE2 receptor-mediated endocytosis in the lower respiratory tract, damaging alveolar epithelial cells that are responsible for producing surfactant to maintain alveolar integrity and pulmonary compliance [240]. Infecting these cells induces an intense innate immune response that compromises alveolar epithelial integrity and increases capillary permeability, resulting in localized endothelial damage [241]. Increased capillary permeability may decrease perfusion and cause endothelial damage, which increases the risk for unresolved clots, a major risk factor for developing chronic PH. All these processes contribute to hypoxia in the pulmonary vasculature, which can also cause PH.

One component of the prolonged pathology following acute SARS-CoV-2 infection may be a persistently injured pulmonary vasculature. Viral antigens may also remain, which drives a chronic inflammation. Identification of the key factors that impairs monocyte and T cell–mediated immunity may uncover mechanisms leading to long COVID since the persistence presence of Spike S1 in nonclassical monocytes [142] and elevated CD8 T cell responses were found in PASC individuals [242]. It is also unclear if treating COVID-19 acutely such as with remdesivir or modulating the immune system in acute disease such as with dexamethasone causes a decrease in the prevalence of long-term symptoms. The clinical studies to date studying immunomodulation in COVID-19 have used short-term endpoints such as progression to more severe disease, or mortality or ventilator-free days out of the first 28. These studies have not investigated late endpoints such as the persistence or delayed development of PH years following acute illness. It is reasonable to assume that if PH were to persist or develop late after acute COVID-19, the prevalence would be higher in those with more severe disease at the peak of their illness. Thus, the clinical benefit observed of therapies in preventing progression to more severe disease may also be avoiding the late, postinfectious complication of PH. Also, acute PH or PH that persist for initial few months as reported recently [125] may not resolve in individuals with coinfections or comorbidities with underlying chronic inflammation and/or subclinical cardiopulmonary diseases.

An additional issue is whether drugs currently used in the treatment of PH and lung fibrosis can benefit patients with PVD associated to long COVID-19. Phosphodiesterase 5 inhibitors, endothelin-1 receptor antagonists, soluble guanylyl cyclase stimulators, prostacyclin analogues, and antifibrotic drugs (pirfenidone and nintedanib) are potentially useful for long COVID-19 patients, but only small and inconclusive clinical trials have been conducted so far [243,244].

The recent progress in the COVID-19-related transgenic animal models [236,245] might be useful in some extent to understand if SARS-CoV2 infection worsens the course or development of experimental PH. For example, we may infect transgenic animals with SARS-CoV2 before or after exposing these to the well-established stimuli of PH such as hypoxia [246], monocrotaline [247], or SUGEN/hypoxia [248] to see if this virus exposure exacerbates the PH-related pathologies.

Another potential method for studying inflammation-mediated alterations in the pulmonary vasculature is vascular organoid models. Blood vessel organoid systems contain endothelial cells and pericytes and can be useful to investigate the drivers of vascular injury due to COVID-19 inflammation [249]. Comparing patient-derived vascular organoid models from patients with long COVID specifically might enable to unravel inflammation-mediated dynamics, which regulates pulmonary vascular remodeling. Additionally, human capillary organoids [249], vascular lung cocultures [250], and pulmonary artery-on-a-chip models [251] can be further adapted to long COVID concept, in order to elucidate the route of inflammation-mediated pulmonary vascular remodeling.
